# Electroporation as an Efficacy Potentiator for Antibiotics With Different Target Sites

**DOI:** 10.3389/fmicb.2021.722232

**Published:** 2021-10-18

**Authors:** Žana Lovšin, Anja Klančnik, Tadej Kotnik

**Affiliations:** ^1^Faculty of Electrical Engineering, University of Ljubljana, Ljubljana, Slovenia; ^2^Biotechnical Faculty, University of Ljubljana, Ljubljana, Slovenia

**Keywords:** electroporation, antibiotics, mode of action, combined antibacterial treatments, wastewater treatment, treatment time

## Abstract

Antibiotic resistance is a global health threat, and there is ample motivation for development of novel antibacterial approaches combining multiple strategies. Electroporation is among the promising complementary techniques – highly optimizable, effective against a broad range of bacteria, and largely impervious to development of resistance. To date, most studies investigating electroporation as an efficacy potentiator for antibacterials used substances permissible in food industry, and only few used clinical antibiotics, as acceptable applications are largely limited to treatment of wastewaters inherently contaminated with such antibiotics. Moreover, most studies have focused mainly on maximal achievable effect, and less on underlying mechanisms. Here, we compare *Escherichia coli* inactivation potentiation rates for three antibiotics with different modes of action: ampicillin (inhibits cell wall synthesis), ciprofloxacin (inhibits DNA replication), and tetracycline (inhibits protein synthesis). We used concentrations for each antibiotic from 0 to 30× its minimum inhibitory concentration, a single 1-ms electric pulse with amplitude from 0 to 20 kV/cm, and post-pulse pre-dilution incubation either absent (≲1 min) or lasting 60 min, 160 min, or 24 h. Our data show that with incubation, potentiation is significant for all three antibiotics, increases consistently with pulse amplitude, and generally also with antibiotic concentration and incubation time. With incubation, potentiation for ampicillin was rather consistently (although with weak statistical significance) superior to both ciprofloxacin and tetracycline: ampicillin was superior to both in 42 of 48 data points, including 7 with significance with respect to both, while at 60- and 160-min incubation, it was superior in 31 of 32 data points, including 6 with significance with respect to both. This suggests that electroporation potentiates wall-targeting antibiotics more than those with intracellular targets, providing motivation for in-depth studies of the relationship between the mode of action of an antibiotic and its potentiation by electroporation. Identification of substances permissible in foods and targeting the cell wall of both Gram-negative and Gram-positive bacteria might provide candidate antibacterials for broad and strong potentiation by electroporation applicable also for food preservation.

## Introduction

Antibiotic resistance is a global health threat associated with increased morbidity, mortality, hospitalization, and healthcare costs (Laxminarayan et al., 2013; Frieri et al., 2017; Klein et al., 2019). There is thus high motivation for development of novel approaches combining multiple antibacterial strategies that have different modes of action (Alexopoulos et al., 2019; Berdejo et al., 2019; Douafer et al., 2019; Juma et al., 2020). Among the promising complementary techniques for such approaches is electroporation, in which exposure of bacteria to short electric pulses of sufficient strength permeabilizes their membranes, thereby facilitating the uptake of diverse compounds, including antibiotics (Garner, 2019). Electroporation is highly optimizable through adjustment of pulse amplitude and duration, reproducible, and effective across a broad range of microorganisms [see Table 1 in Kotnik et al. (2015)]. Moreover, due to the physical nature of the main underlying mechanism – formation of aqueous pores in the membranes (Kotnik et al., 2019) – bacteria cannot develop resistance against it.

Electroporation alone can also cause bacterial death, but the levels of inactivation typically achieved are insufficient for general stand-alone use, providing motivation for development of synergistic treatments (Martín-Belloso and Sobrino-López, 2011; Berdejo et al., 2019). For food and beverage preservation, the combination of electroporation with antibacterials has been shown to decrease required pulse amplitudes and increase achievable inactivation rates (Berdejo et al., 2019). However, the range of antibacterials permissible for such applications is in practice limited to substances that either occur also naturally in food (e.g., acetic acid, citric acid, lactic acid, cinnamaldehyde, and linalool) or are registered as food additives (e.g., nisin, triethyl citrate).

For any application outside clinical and veterinary medicine, addition of antibiotics is generally problematic due to the involved health risks and/or the resulting environmental burden. However, wastewaters from hospitals and livestock farms are already inherently contaminated with clinical and veterinary antibiotics, respectively (Diwan et al., 2013; Kim et al., 2020), as well as with a broad range of bacteria including strains resistant to different antibiotics (Hocquet et al., 2016). As a result, downstream wastewater treatment plants where all these antibiotics and bacteria accumulate become hotbeds for cross-acquisition of bacterial antibiotic resistance and for release of resistance genes into the environment, where they can further contribute to the spread of resistance (Rizzo et al., 2013). The ensuing problem is further aggravated by hospitals’ use and subsequent release into wastewater of novel and last-resort antibiotics, for which it is of paramount importance to retain the absence of bacterial resistance.

These problems are increasingly recognized as being critical both for human health and for the environment, and thus approaches to reduce the concentration of viable bacteria, antibiotics, and/or antibiotic resistance genes – preferably all three – in wastewaters are now being proposed and tested. For on-site treatment of wastewaters prior to their release into the communal sewage, various designs have been proposed based on ozonation, ultraviolet light, and/or microalgal biodegradation (Paulus et al., 2019; Leng et al., 2020).

With wastewaters already containing various bacteria and antibiotics, methods that render the bacterial envelope permeable, thus facilitating the permeation of antibiotics into the bacteria and potentiating their efficacy, emerge as straightforward candidates for the first “line of attack.” Once the bacterial load is sufficiently reduced, subsequent treatment can focus on degradation/removal of the antibiotics and the bacterial resistance genes. As permeabilization based on additives (e.g., detergents, enzymes, and microbeads) would cause additional pollution of the treated wastewater, the acceptable options are limited to green techniques, such as ultrasonication, freeze-thawing, and electroporation. Among these, electroporation is most widely recognized and used due to its general effectiveness, efficiency, and applicability to a broad range of microorganisms (Aune and Aachmann, 2010; Kotnik et al., 2015; Eleršek et al., 2020). Furthermore, in contrast to the mainstream water treatment method of chlorination, which in wastewaters can create genotoxic adsorbable organic chlorides (Emmanuel et al., 2004), delivery of electric pulses does not increase wastewater genotoxicity even at highest amplitudes and durations used in practice for electroporation (Gusbeth et al., 2009).

To date, the majority of studies that have combined electroporation and antibacterials [see Table 2 in Garner (2019)] have focused on substances permissible in food and beverage processing. Still, five recent studies have quantified the potentiation of inactivation rates for combination of electroporation and clinical antibiotics (Korem et al., 2018; Novickij et al., 2018a; Vadlamani et al., 2018, 2020; Rubin et al., 2019), with their exposure parameters and results summarized in Table 1. Two further studies (Kuyukina et al., 2020; Martens et al., 2020) used the disk diffusion test to study electroporation-induced increase in susceptibility of *Rhodococcus ruber* to five and *Escherichia coli* to four different antibiotics, at increasing concentrations, and confirmed that such susceptibility increases are generally achievable.

**TABLE 1:** Summary of the five reports on combined antibacterial use of clinical antibiotics and electroporation.

Study	Bacteria	Antibiotic		Pulse parameters				Maximum effect (IR = inactivation rate; VR = viability rate)
		Type	Concentration (μg/mL)	Pulse duration (μs)	Number of pulses	Pulse amplitude (kV/cm)	Repetition frequency (Hz)	
Novickij et al., 2018a	*Staphylococcus aureus* (MRSA)	Ciprofloxacin	1, 10, 100, and 1,000	100	8	5, 10, 15, 20	1,000	<25% VR @ 20 kV/cm, 1,000 μg/ml
		Doxycycline	1, 10, 100, and 1,000					<3% VR @ 20 kV/cm, 1,000 μg/ml
		Gentamicin	1, 10, 100, and 1,000					<1% VR @ 15 kV/cm, 10 μg/ml
		Sulfamethoxazole	1, 10, 100, and 1,000					<5% VR @ 20 kV/cm, ≥1 μg/ml
		Vancomycin	1, 10, 100, and 1,000					<16% VR @ 20 kV/cm, 1,000 μg/ml
Rubin et al., 2019	*Pseudomonas aeruginosa*	Mix of penicillin G (500–5,000 U/mL), streptomycin (0.5–5 mg/mL), nystatin (antimycotic, 62.5–625 U/mL)		60	200	≤∼3.1	2.8	Same IR @ ∼30–45% lower pulse amplitude
	*Staphylococcus epidermidis*					≤∼3.8		Same IR @ ∼8–13% lower pulse amplitude
Korem et al., 2018	*Staphylococcus aureus* (oxacillin-sensitive)	Oxacillin	0.5×, 1×, 2 × MIC (MIC = 0.38 μg/mL)	100	∼50 pulses 2.0 kV and ∼50 pulses 1.5 kV		1	No detectable VR @ ≥0.5 × MIC
Vadlamani et al., 2018	*Staphylococcus aureus*	Tobramycin	0.2, 2, and 20	0.3	1,000 pulses 20 kV/cm, 445 pulses 30 kV/cm, or 250 pulses 40 kV/cm		1	>5.5 log_10_ IR @ 40 kV/cm, 20 μg/ml
	*Escherichia coli*							>4.2 log_10_ IR @ 30 kV/cm, 20 μg/ml
	*Staphylococcus aureus*	Rifampicin	0.2, 2, 20					>5.2 log_10_ IR @ 30 kV/cm, 20 μg/ml
	*Escherichia coli*							>8.5 log_10_ IR @ 30 kV/cm, 20 μg/ml
Vadlamani et al., 2020	*Staphylococcus aureus* (MRSA)	Mupirocin	2, 20	0.3	500 pulses 20 kV/cm or 222 pulses 30 kV/cm		1	≤6.5 log_10_ IR
	*Escherichia coli*							≤4.5 log_10_ IR
	*Pseudomonas aeruginosa*							≤1.3 log_10_ IR
	*Staphylococcus aureus* (MRSA)	Rifampicin	2, 20					≤6.3 log_10_ IR
	*Escherichia coli*							≤6.4 log_10_ IR
	*Pseudomonas aeruginosa*							≤2.1 log_10_ IR
	*Staphylococcus aureus* (MRSA)	Erythromycin	2, 20					≤4.8 log_10_ IR
	*Escherichia coli*							≤4.4 log_10_ IR
	*Pseudomonas aeruginosa*							≤1.0 log_10_ IR
	*Staphylococcus aureus* (MRSA)	Vancomycin	2, 20					≤5.3 log_10_ IR
	*Escherichia coli*							≤4.5 log_10_ IR
	*Pseudomonas aeruginosa*							≤1.4 log_10_ IR

**TABLE 2:** Antibiotics and concentrations used in the experiment.

Antibiotic	Concentration (μg/mL)			
	MIC	3 × MIC	10 × MIC	30 × MIC
Ampicillin	30	90	300	900
Ciprofloxacin	0.025	0.075	0.25	0.75
Tetracycline	2.0	6.0	20	60

*MIC, minimal inhibitory concentration.*

As summarized in Table 1, the studies to date have shown that electroporation generally potentiates the efficacy of antibiotics, and that potentiation is achievable whether the antibiotic targets the synthesis of nucleic acids (ciprofloxacin, rifampicin), proteins (doxycycline, erythromycin, gentamicin, mupirocin, streptomycin, and tobramycin), or the cell wall (oxacillin, penicillin G, and vancomycin). Still, a systematic evaluation of the possible dependence of this potentiation on the antibiotic’s mode of action is lacking, yet needed for a broader understanding and recognition of electroporation as an effective potentiator of antibiotics, and for its implementation in practice. Moreover, for cross-comparison of potentiation rates achievable for different antibiotics, the most informative approach is to proceed for each investigated antibiotic from its minimum inhibitory concentration (MIC), which has to date only been used in one study to investigate a single antibiotic (Korem et al., 2018).

Also, where stated in these previous studies, the post-pulse incubation times before diluting out of the antibiotics have been as long as 24 h (Novickij et al., 2018a; Kuyukina et al., 2020; Martens et al., 2020). Implementation of such a long incubation for on-site hospital wastewater treatment would require a reservoir in which the undiluted antibiotic concentration would be retained until release into the communal sewage (and thus dilution), and the required reservoir volume would likely be prohibitive. Lastly, in these previous studies, bacteria were subjected to at least 8 and up to 1,000 consecutive pulses per treatment, which maximized the effect, but at the cost of adding to the list of parameters (bacterial strain, antibiotic type, antibiotic concentration, pulse duration, pulse amplitude, and post-treatment incubation time) two more – the number of pulses and their repetition frequency.

Here, we compared the *E. coli* inactivation potentiation by electroporation for three antibiotics with different modes of action: ampicillin (inhibits cell wall synthesis), ciprofloxacin (inhibits DNA replication), and tetracycline (inhibits protein synthesis). The inactivation rates were investigated at antibiotic concentrations from the MIC to 30-fold the MIC, exposure to a single 1-ms electric pulse with amplitudes from 5 to 20 kV/cm, and a post-pulse pre-dilution incubation from ≲1 min to 24 h.

## Materials and Methods

### Bacterial Strain and Growth Conditions

To exclude the effect of resistance and virulence genes, the non-resistant and non-pathogenic *E. coli* K12 ER1821 strain was used (New England BioLabs, Ipswitch, MA, United States), and was propagated according to the protocol of the supplier. The cells were cultured in lysogeny broth rich medium (Sigma-Aldrich, St. Louis, MO, United States) at 37°C, with agitation. The growth curve was measured from a starting culture with optical density at 600 nm (OD_600_) of 0.01, and the middle exponential phase was determined at 3.5 h of incubation.

### Antibiotics and Minimum Inhibitory Concentration Determination

Three antibiotics with different modes of action were used in this study: (i) ampicillin (#A9518; Sigma-Aldrich), which inhibits cell wall synthesis by binding to bacterial penicillin-binding protein transpeptidases, thus preventing them from catalyzing cross-linking of peptidoglycan chains (Wright, 1999); (ii) ciprofloxacin (#17850; Sigma-Aldrich), which inhibits DNA replication by binding to and thus blocking bacterial DNA gyrase and topoisomerase IV (Madurga et al., 2008); and (iii) tetracycline (#T3383; Sigma-Aldrich), which inhibits protein synthesis by preventing the attachment of aminoacyl-tRNA to the A-site of the bacterial 30S ribosomal subunit (Chopra and Roberts, 2001).

To allow for cross-comparisons of the potentiation achievable for each of these antibiotics, the minimum inhibitory concentration (MIC) for each antibiotic was determined against the *E. coli* strain used, as the lowest concentration of the antibiotic that inhibited visible growth of the *E. coli* during the incubation. This is to be distinguished from the minimum bactericidal concentration, MBC, which is higher and is defined as the lowest concentration of an antibiotic that kills at least 99.9% of the bacteria. The standard protocol of agar dilution and overnight incubation was followed for MIC determination (Andrews, 2001), with the MIC values so determined given in section “Antibiotics Minimum Inhibitory Concentrations.” Experiments were then carried out at the antibiotic concentrations corresponding to MIC, 3 × MIC, 10 × MIC, and 30 × MIC, with the multiples of MIC used to compensate for the shorter post-pulse pre-dilution incubation times used in these experiments compared to the overnight incubation used for MIC determination.

### Sample Preparation

Overnight *E. coli* cultures were initiated by inoculation of one colony from lysogeny broth agar plate to 50 mL lysogeny broth, with overnight incubation at 37°C, with agitation. The following morning OD_600_ was measured, and fresh 250 mL lysogeny broth was inoculated for the starting OD_600_ of 0.01. The cultures were grown to the middle exponential growth phase, which occurred at 3.5 h at 37°C, with agitation. The cells were then centrifuged, washed with 250 mM sucrose, centrifuged again, and resuspended in 16 mL 250 mM sucrose.

### Treatment: Antibiotic Concentrations, Electric Pulse Amplitudes, and Post-Pulse Pre-Dilution Incubation Times

For each antibiotic, the concentrations used were 0 (no antibiotic), MIC, 3 × MIC, 10 × MIC, and 30 × MIC. For the electric pulse, the amplitudes used were 0 (no pulse delivery), 5, 10, 15, and 20 kV/cm. For post-pulse pre-dilution incubation at room temperature with the same antibiotic at the same concentration as in the treatment, the incubation times used were ≲1 min (dilution right after pulse delivery), 60 min, 160 min, and 24 h.

For each combination of parameters, the experiments were performed three times on different days, with altered order in which the parameters were varied for each of three repetitions of the experiment. The treatment with no antibiotic and no pulse delivery was considered as the control.

**FIGURE 1:**
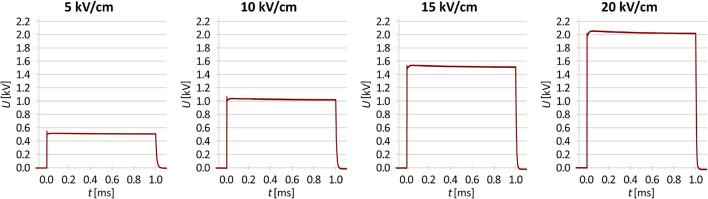
The actual time courses of the voltage delivered in this study by the 5, 10, 15, and 20 kV/cm pulse.

A 3-mL volume of prepared washed culture was added to 3 mL of 250 mM sucrose (for control and electroporation-only experiments), or to 3 mL of 250 mM sucrose supplemented with an antibiotic at the final concentration required (i.e., MIC or multiples thereof) as stated above. Electroporation was performed using the HVP-VG square wave pulse generator (IGEA, Carpi, Italy). The samples (140 μL) were placed between two parallel stainless-steel electrodes with a 1-mm gap, and a single 1-ms rectangular electric pulse was delivered. The voltages applied were 500, 1,000, 1,500, and 2,000 V, for electric pulse amplitude (i.e., voltage-to-distance ratio) of 5, 10, 15, and 20 kV/cm, respectively, with the actual time courses of the voltage for each pulse amplitude as measured with LeCroy HDO4104A oscilloscope and HVD3206A voltage probe (Teledyne Technologies, Thousand Oaks, CA, United States) shown in Figure 1. Then, a 100-μL volume was taken from each sample, and mixed with 100 μL of either lysogeny broth (for control and electroporation-only experiments) or lysogeny broth supplemented with the antibiotic at the final concentration required for the post-pulse pre-dilution incubation.

### Determination of Inactivation Rate

After the treatment and the post-treatment incubation, the *E. coli* samples were serially diluted in 0.9% NaCl, and for each dilution, three drops of 10 μL were plated on a lysogeny broth agar plate. After the drops dried, the plates were incubated overnight at 37°C. The *E. coli* counts were recorded for each dilution (colony counts from 3 to 30), and the colony forming units (CFU)/mL were calculated from the mean number of colonies (mean of the three drops). The *E. coli* inactivation rates were calculated as −log_10_(*N*/*N*_0_), where *N* is the *E. coli* CFU/mL of the sample, and *N*_0_ is the *E. coli* CFU/mL of the control (log_10_ will henceforth be referred to as log).

### Statistical Analysis

**FIGURE 2:**
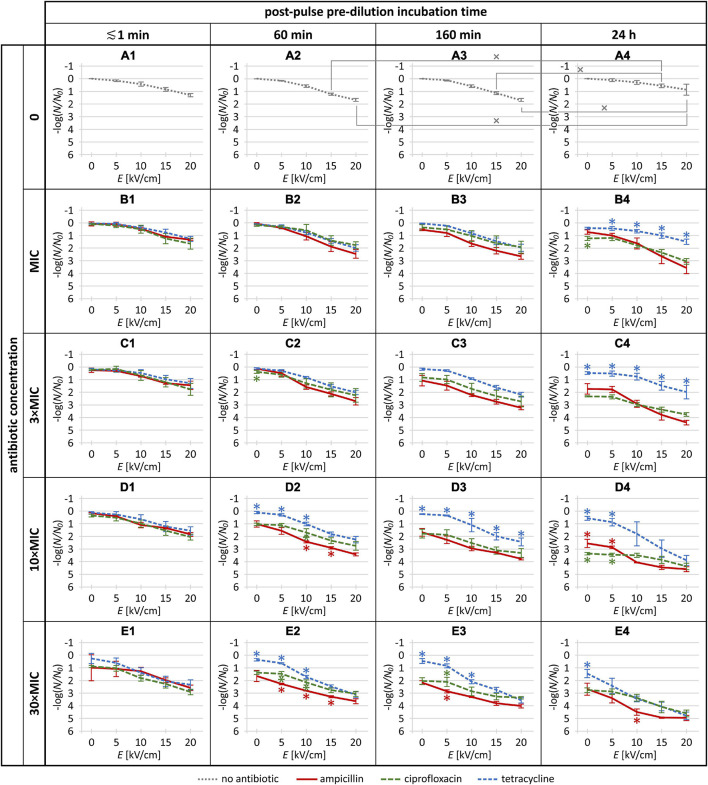
The *Escherichia coli* inactivation rates in the absence of antibiotics (gray dotted) and for each of the three investigated antibiotics (red solid: ampicillin; green long-dashed: ciprofloxacin; blue short-dashed: tetracycline) at the MIC, 3 × MIC, 10 × MIC, and 30 × MIC, combined with a single 1-ms electric pulse at amplitude (*E*) of 0 (i.e., no pulse), 5, 10, 15, and 20 kV/cm. The plot labels **(A1–E4)** are provided to facilitate the referencing of individual plots in the body text. Each data point is represented as mean ± standard deviation of 3 replicates. In panels **(A1–A4)**, tilted crosses (×) mark pairs of data points where the inactivation rate achieved with the same pulse amplitude was statistically significantly different (*p* < 0.05) for different incubation times. In panels **(B1–E4)**, asterisks (*) mark data points where the inactivation rate was statistically significantly different (*p* < 0.05) for one antibiotic versus both others (red *: ampicillin vs. both ciprofloxacin and tetracycline; green *: ciprofloxacin vs. both ampicillin and tetracycline; blue ^∗^: tetracycline vs. both ampicillin and ciprofloxacin). Raw data and further statistical analysis are provided in the Supplementary Material.

The experiments were repeated three times on different days for each antibiotic, and the treatment data were normalized to the control (i.e., sample with no antibiotic and no pulse delivery) and expressed as mean ± standard deviation. The data were post-processed in R Commander 2.6 (developed by John Fox at McMaster University, Hamilton, Canada, and available under the GNU General Public License). To compare the effects of the three antibiotics, one-way analysis of variance was used (ANOVA; *p* < 0.05) for each combination of electric pulse amplitude, antibiotic concentration, and post-pulse pre-dilution incubation time. Tukey’s HSD multiple comparison test for evaluation of the difference was used when ANOVA indicated a statistically significant difference (*p* < 0.05). In Figure 2, asterisks indicate data points where the effect with one antibiotic was statistically significantly different from each of the other two (e.g., a data point for ampicillin was assigned an asterisk if it was significantly different from both ciprofloxacin and tetracycline, and similarly with data points for the other two antibiotics).

## Results

### Antibiotics Minimum Inhibitory Concentrations

The minimum inhibitory concentrations (MICs) against *E. coli* were determined initially, using the standard protocol of agar dilution and overnight incubation of the *E. coli* with each antibiotic. The MICs were 30 μg/mL for ampicillin, 0.025 μg/mL for ciprofloxacin, and 2 μg/mL for tetracycline. As to compensate for the much shorter incubation times used in most of our subsequent experiments combining antibiotics with electroporation, we performed these at MIC as well as at 3 × MIC, 10 × MIC, and 30 × MIC, with corresponding concentrations in μg/mL given in Table 2.

Of note, even the highest antibiotic concentrations used here (i.e., 30 × MIC, as determined in overnight *E. coli* cultures) were not bactericidal even after 24 h of incubation with each of the antibiotics. This was because while the growth of *E. coli* cells was inhibited throughout the incubation, after the transfer to rich growth medium they recovered (e.g., see Figure 2E4).

### Inactivation With Antibiotics and Electroporation

The *E. coli* inactivation rates obtained in the absence of antibiotics and for each of the three antibiotics at the MIC, 3 × MIC, 10 × MIC, and 30 × MIC, combined with a single 1-ms electric pulse at amplitude of 0 (no pulse), 5, 10, 15, and 20 kV/cm, are presented in Figure 2, with the raw experimental data and further statistical analysis provided in the Supplementary Material.

#### Electroporation Treatment Alone

When the *E. coli* cells were treated with electroporation alone (Figures 2A1–A4), 5 kV/cm amplitude had only a minor and statistically non-significant effect on inactivation rate (≲0.2 log) regardless of post-pulse incubation time, while as the amplitude was increased (to 10, 15, and 20 kV/cm), the inactivation rate gradually increased. This was expected, as empirically for most bacteria, a single 1-ms electric pulse with an amplitude of ∼5 kV/cm causes only mild and reversible electroporation, while amplitudes of 10, 15, and 20 kV/cm are roughly at the lower end, middle, and higher end, respectively, of the range of non-thermal irreversible electroporation [e.g., see Figure 1A in Kotnik et al. (2015)]. More precisely, for each of the four post-pulse incubation times, the maximum inactivation rate was obtained at the maximum amplitude used, 20 kV/cm, reaching ∼1.3 log for the ≲1-min post-pulse incubation, ∼1.7 log for 60- and 160-min post-pulse incubations, and ∼0.9 log for 24-h post-pulse incubation. This relative recovery for the longest post-pulse incubation indicates that in the absence of antibiotics, the *E. coli* were gradually starting to grow and proliferate again.

We note here that in comparison to these bacterial inactivation rates obtained by electroporation alone, many studies report much higher rates, for two reasons. First, most studies have aimed for the maximum achievable effect, and have thus applied tens, hundreds, or even up to 1,000 consecutive pulses per treatment (see Table 1), while our aim was to investigate whether efficacy potentiation by electroporation for an antibiotic depends on the latter’s mode of action; thus we used a single pulse to keep the analysis of the investigated dependence straightforward. Second, again to maximize the inactivation rates, some studies have applied pulse amplitudes of 30 or even 40 kV/cm, while here we used amplitudes up to 20 kV/cm, to assure that the contribution of electroporation was not entangled with those of electric arcing (with accompanying mechanical shockwaves and ultraviolet light) and thermal damage that can occur at higher pulse amplitudes.

#### Electroporation as an Efficacy Potentiator for Antibiotics

As stated previously, our main aim was to investigate whether efficacy potentiation by electroporation for an antibiotic depends on its mode of action. However, as outlined in the penultimate paragraph of the Introduction, from the perspective of limitations in practical applications, a post-pulse pre-dilution incubation time as long as 24 h is prohibitive from the aspect of the required reservoir volume, so we first tested whether this incubation time can perhaps be eliminated altogether, or at least shortened considerably. Thus, we initially considered whether reasonable potentiation can be achieved even with dilution performed right after the treatment (after ≲1 min incubation), and then we focused on the roles of (longer) post-pulse pre-dilution incubation time and of antibiotic concentrations.

##### Potentiation with dilution right after pulse delivery

With the antibiotic dilution right after the electroporation pulse treatment (i.e., ≲1 min incubation), no significant difference was seen between ampicillin, tetracycline, and ciprofloxacin (Figures 2B1–E1). With the lowest pulse amplitude (5 kV/cm), the increase in *E. coli* inactivation rate was small, particularly for the antibiotic concentrations up to 10 × MIC, and although at 30 × MIC (Figure 2E1) ampicillin and ciprofloxacin appeared more effective than tetracycline, the differences did not reach statistical significance. These data indicate that irrespective of their mode of action, efficacy of antibiotics is not significantly potentiated by electroporation when the antibiotic is diluted out within a minute or less after pulse delivery.

##### Potentiation with dilution after post-pulse incubation of 60 min, 160 min, and 24 h

When post-pulse pre-dilution incubation time was increased to 60 min, 160 min, and 24 h, relative to the ≲1 min incubation the potentiation also increased for each antibiotic. Specifically, three observed parametric dependences can be inferred.

First, the antibiotic potentiation consistently increased with the increase of pulse amplitude (i.e., in each of the panels of Figure 2, each of the curves has a downward slope).

Second, the antibiotic potentiation generally also increased with the increase of antibiotic concentrations at each of these three post-pulse incubation times (Figures 2B2↘ **C2** ↘ **D2** ↘ **E2**; **B3** ↘ **C3** ↘ **D3** ↘ **E3**; **B4** ↘ **C4** ↘ **D4** ↘ **E4**). A single exception here was for ciprofloxacin at 24-h post-pulse incubation for the 5 kV/cm pulse, with indication of lower *E. coli* inactivation for 30 × MIC compared to 10 × MIC, although this did not reach statistical significance (i.e., in Figure 2, the second data point of the green curve is higher in **E4** than in **D4**).

Third, the antibiotic potentiation generally also increased with the increase of the post-pulse incubation time at each of the four antibiotic concentrations (Figures 2B2↘ **B3** ↘ **B4**; **C2** ↘ **C3** ↘ **C4**; **D2** ↘ **D3** ↘ **D4**; **E2** ↘ **E3** ↘ **E4**). Here the exception was tetracycline at MIC and 3 × MIC, with indications of lower *E. coli* inactivation for 24-h than for 60- or 160-min post-pulse incubations; however, again, none of these reached statistical significance (i.e., in Figure 2, the blue curve is partly higher in **B4** vs. **B2** and **B3**, and partly higher in **C4** vs. **C2** and **C3**).

As seen in Figure 2, in many data points the differences did not reach statistical significance, although we note, as elaborated in section “Statistical Analysis,” that we only marked by an asterisk those data points for an antibiotic for which its *E. coli* inactivation rate differed statistically significantly from both other antibiotics. Despite this, there was a relatively clear general trend of superior inactivation rates for ampicillin compared to ciprofloxacin and tetracycline (i.e., in Figure 2, the red curves are rather consistently below the green and blue ones), particularly for the intermediate post-pulse incubation times of 60 and 160 min. On the opposite end, potentiation of *E. coli* inactivation rates was generally the weakest with tetracycline, although at the highest concentrations combined with the highest pulse amplitude this was less pronounced (Figures 2D4,E2–E4).

In quantitative terms, all the data presented in Figure 2 for the combination of an antibiotic, electric pulse, and post-pulse incubation (60 min, 160 min, or 24 h), can be summarized into two aspects.

First, for all three incubation times, i.e., considering in Figures 2B2–E4 on all the curves the 48 data points for pulse amplitudes from 5 to 20 kV/cm, for ampicillin 42 of these data points were superior to both ciprofloxacin and tetracycline, of which 7 reached statistical significance with respect to both.

Second, if we restrict this analysis to only the 60- and 160-min post-pulse incubations, of the 32 data points that thus remain on the relevant curves (Figures 2B2–E3), for ampicillin 31 of these data points were superior to both ciprofloxacin and tetracycline, of which 6 reached statistical significance with respect to both. The only exception here was for 3 × MIC with 5 kV/cm pulse and 60 min post-pulse incubation (Figure 2C2, second data point), where ampicillin appeared to be inferior to ciprofloxacin but still superior to tetracycline, although these apparent differences did not reach statistical significance.

## Discussion

Considering the different target sites of the three antibiotics, the rather consistently superior efficacy potentiation for ampicillin can be explained by its easier access to its particular target: the bacterial cell wall. Namely, ampicillin targets the sites of peptidoglycan chains cross-linkage by inhibiting the transpeptidase enzyme that catalyzes this cross-linkage, which destabilizes the local structure and the cell wall as a whole. Thus, for ampicillin to exhibit its antibacterial activity, permeation-enabling disruption of the inner (cytoplasmic) bacterial membrane is not required, in contrast to both ciprofloxacin and tetracycline that have intracellular targets (the sites of DNA replication and protein synthesis, respectively), for access to which they must permeate through all the layers of the bacterial envelope.

The data for dilution right after pulse delivery (i.e., ≲1 min incubation) imply, however, that even for ampicillin, substantial efficacy potentiation requires time, with *E. coli* inactivation rates improved by an order of magnitude when the dilution of the antibiotic was delayed by 60 or 160 min, while the 24-h delay resulted in more sporadic further improvements and mostly at the highest pulse amplitudes. This suggests that shortening the post-pulse incubation time with the antibiotic from 24 h (as used in many previous studies) to one or several hours is feasible, with proportionally reduced reservoir volume required in applications for wastewater treatment.

The small and sporadic further improvements in inactivation rates with the 24-h incubation are most likely due to the physiological uptake of the antibiotics, which would occur even without electroporation by gradual permeation through the intact bacterial envelope. This is reflected in the small but rather consistent improvement of the inactivation rates for all three antibiotics at 0 kV/cm (i.e., without electric pulse delivery) at 24-h incubation compared to 60- or 160-min incubation, which is detectable also for MIC and 3 × MIC, but is more evident for 10 × MIC and even more so for 30 × MIC. The role of physiological permeation on longer time scales is also consistent with the empirical fact that in medical and veterinary therapies with an antibiotic alone, its concentration must be maintained at a suprainhibitory level for days, and for some infections even for weeks to achieve an effective outcome (Wormser et al., 2004).

Regarding the generally weakest potentiation for tetracycline, we note that of the three antibiotics used in our study, tetracycline has the highest molecular weight (444 g/mol, vs. 331 and 349 g/mol for ciprofloxacin and ampicillin, respectively) and therefore likely requires stronger and/or more extensive electroporation for similarly potentiated permeation into bacteria. However, we stop short of postulating this as the main reason for the relatively inferior *E. coli* inactivation rates observed here for tetracycline compared to both ampicillin and ciprofloxacin.

Our finding that for a wall-targeting antibiotic, the efficacy against *E. coli* can be potentiated by electroporation to a greater extent – and/or more readily – compared to two antibiotics that target intracellular sites, is also in empirical agreement with findings from a recent study of Kuyukina et al. (2020). Although their study did not focus on the role of the antibiotic target site, for post-pulse incubation times up of to 240 min they found generally superior antibiotic potentiation by electroporation for benzylpenicillin and cefazolin (which also target cell wall synthesis) compared to gentamicin, kanamycin, and neomycin (which target protein synthesis). This is particularly relevant for the more general validity of the thesis that electroporation provides superior potentiation for antibiotics that target the cell wall compared to those with intracellular targets, as *E. coli* (used in the present study) is Gram-negative, while *Rhodococcus ruber* (used by Kuyukina and colleagues) is Gram-positive, and thus the structure of their envelope differs significantly.

However, there are still some obvious and possibly other unforeseen obstacles for the application of this finding in practice. Adding antibiotics is universally problematic in terms of the resulting environmental burden, and in many applications also from the resulting risks to human health. Conversely, the applications utilizing the inherently present antibiotics, such as treatment of wastewaters from hospitals and livestock farms, are dependent on the persistently fluctuating compositions and concentrations of antibiotics, which are also generally well below their MICs (Martinez, 2009; Diwan et al., 2013; Cheng et al., 2020). Thus, although the results presented here show that at the MIC and multiples thereof, a post-pulse incubation time of 1 or 2 h may be sufficient for substantial (∼3–4 log) inactivation rates, this may not be true for the antibiotic concentrations that occur inherently in such wastewaters.

For use in clinical or veterinary applications, and in general for combining antibiotics with electroporation against bacterial infections of eukaryotic organisms, a major and perhaps largely unsurmountable obstacle lies in the fact that most eukaryotic cells are an order of magnitude larger than bacteria. Since the transmembrane voltage induced by exposing a cell to an electric pulse of a fixed amplitude (electric field strength, that can be approximated by the voltage-to-distance ratio) is proportional to the cell size (Pauly and Schwan, 1959; Kotnik et al., 1998; Kotnik and Miklavčič, 2000), a significantly higher transmembrane voltage is induced by the same pulse on eukaryotic cells than on bacteria. As the intensity of electroporation is strongly correlated to the induced transmembrane voltage (Kotnik et al., 2010), the application of electroporating pulses to a eukaryotic tissue infected with bacteria, or in general to a mix of eukaryotic cells and bacteria, will typically result in extensive damage to the eukaryotic tissue (through irreversible electroporation) before achieving electroporation of bacteria.

For use in food industry, the range of permissible antibacterials is limited to those that either occur naturally in foods or are approved as food additives, but if superior potentiation by electroporation for substances targeting the bacterial cell wall holds generally true for antibacterials, applications for food and beverage preservation can (re)focus on those among the permissible substances that target the wall. Currently, one such substance widely recognized as targeting the wall is nisin (Malanovic and Lohner, 2016; Modugno et al., 2018), and there is at least one report of its potentiation by electroporation, achieving moderate (∼2–3 log) inactivation rates against *E. coli* (Novickij et al., 2018b). However, at least one study found no potentiating effect of electroporation for nisin against either *E. coli* or *Salmonella typhimurium* (Saldaña et al., 2012), while the efficacy of nisin alone is largely limited to Gram-positive bacteria (Asaduzzaman and Sonomoto, 2009) and can only be extended to Gram-negative bacteria by artificially modifying the nisin molecule (Field et al., 2015; Zhou et al., 2016) or by binding nanocomposites to it (Vukomanović et al., 2017). There is thus ample motivation for systematic search and identification of antibacterials that are permissible in foods and target the cell wall of Gram-negative as well as Gram-positive bacteria, as this class of compounds should provide optimal candidates for broad and strong potentiation by electroporation applicable also in food and beverage preservation.

## Conclusion

For the understanding of the dependence of the antibiotic efficacy potentiation by electroporation on the antibiotic’s target site, our results presented above suggest that for antibiotics targeting the bacterial cell wall, this potentiation can be higher than for antibiotics with intracellular targets. For broader testing and a deeper understanding of this thesis, further studies are needed, performed with a broader range of antibiotics and on a broader range of bacteria, including comparisons for antibiotic-sensitive vs. antibiotic-resistant strains, and for bacteria in different growth stages. Identification of substances permissible in foods and targeting the cell wall of both Gram negative and Gram positive bacteria might provide candidate antibacterials for broad and strong potentiation by electroporation applicable also in food industry.

## Data Availability Statement

The original contributions presented in the study are included in the article/Supplementary Material, further inquiries can be directed to the corresponding author.

## Author Contributions

ŽL set up the experimental plan, conducted the experiments, processed, analyzed, interpreted the results, and wrote part of the manuscript. TK set up the concept of the experiments, supervised the work, interpreted the results, and wrote part of the manuscript. AK wrote part of the manuscript. All authors have reviewed and approved the final manuscript.

## Conflict of Interest

The authors declare that the research was conducted in the absence of any commercial or financial relationships that could be construed as a potential conflict of interest.

## Publisher’s Note

All claims expressed in this article are solely those of the authors and do not necessarily represent those of their affiliated organizations, or those of the publisher, the editors and the reviewers. Any product that may be evaluated in this article, or claim that may be made by its manufacturer, is not guaranteed or endorsed by the publisher.
